# Collaborative Gameplay for Children’s Self-Efficacy in Computational Thinking: Qualitative Cross-Sectional Design Science Research Case Study

**DOI:** 10.2196/94416

**Published:** 2026-09-09

**Authors:** Valéria Moreira Pinto, Mariana Seiça, Licinio Roque

**Affiliations:** 1 Institute for Interdisciplinary Research, CISUC/LASI – Centre for Informatics and Systems of the University of Coimbra, University of Coimbra, Casa Costa Alemão, R. Dom Francisco de Lemos, Coimbra, 3030-789, Portugal, 351 239247800; 2 Department of Informatics Engineering, CISUC/LASI – Centre for Informatics and Systems of the University of Coimbra, University of Coimbra, Coimbra, Portugal

**Keywords:** cooperative gameplay, self-efficacy, computational thinking, game design, design principles, collaborative

## Abstract

**Background:**

Despite science, technology, engineering, and mathematics (STEM) fields driving socioeconomic development and career prospects, they continue to struggle with low enrollment and high dropout rates. An important predictor of academic performance and career persistence is self-efficacy, which is the belief in one’s ability to succeed. Research indicates that collaboration improves learning outcomes and is associated with improved STEM motivation. Computational thinking (CT) is also increasingly valued in STEM education due to its reliance on computational tools and problem-solving processes. Early exposure to STEM content in a child’s educational journey may thus be a key opportunity for shaping their STEM self-efficacy and future career path.

**Objective:**

This study aimed to explore how a collaborative, playful learning experience influences self-efficacy in CT skills among children. This study followed an iterative Design Science Research (DSR) approach. In this study, we present the first iteration of the DSR process, focused on analyzing and retrieving design principles for exploring how collaborative gameplay may support self-efficacy in CT as a leverage for STEM education.

**Methods:**

For this study, a low-resolution study tabletop game, MeerPlay, was conceived as our main instrument to perform 5 gameplay rehearsal sessions with 14 children, aged between 6 and 11 years. Data were collected following a qualitative content analysis methodology, using video recordings of the sessions and individual semistructured interviews. The content analysis followed an axial coding approach, focused on retrieving evidence of collaborative behaviors that might have supported self-efficacy in CT during gameplay.

**Results:**

Results suggest that collaboration enables verbal persuasion, vicarious experience, and mutual problem-solving, which are important factors in self-efficacy development. The players engaged in a collaborative dialogue and strategic cooperative gameplay by sharing strategies and ideas, creating an environment of mutual support and encouragement. Participants gradually developed skills to overcome the game’s challenges, perform new gameplay actions, and work together toward accomplishing the game’s goal, thus supporting self-efficacy in CT challenges.

**Conclusions:**

We propose a set of game design principles for collaborative gameplay: (1) a shared player panel to jointly define the characters’ actions, (2) conjoined game actions to achieve the overall objective, (3) shared in-game elements and actions, (4) shared dice-roll values to incite collaborative movements, and (5) community evolution and fate across time, promoting a sense of joint victory, loss, and reward. We introduce an innovative methodological approach to study how the design elements influence collaborative interactions supporting self-efficacy. Framing STEM promotion as a sociotechnical wicked problem*,* we hope these design insights will be expanded in serious game design and other technical skill development contexts (eg, engineering, health, and education), potentially shaping academic interests and career paths in future generations.

## Introduction

### Background

With rapid technological advancement, it is crucial to encourage scientific curiosity and opportunities in the fields of science, technology, engineering, and mathematics (STEM) to address the growing demand for skilled individuals in these sectors [1-5]. Despite the availability of STEM programs and favorable labor market outcomes, engineering fields have a disproportionately low number of students [2] and a high dropout rate [4,6], with many switching to non-STEM fields [7-9]. It is thus essential to develop strategies to motivate and attract more students to these fields [3,7]. The goal of this study was to explore the relationship between self-efficacy and computational thinking (CT) through a game-based environment to study the effects of collaborative dynamics in developing hands-on learning, problem-solving, and teamwork (key skills in STEM careers) [10,11].

### Review of Relevant Scholarship

#### Sources of Self-Efficacy, Vocational Choices, and Games

Bandura [12] defines self-efficacy as an individual’s belief in their ability to “successfully execute the behavior required to produce the outcomes” (page 193), with this belief determining “how much effort will be expended, and how long it will be sustained in the face of obstacles and aversive experiences” (page 191), thus influencing personal choices, motivation, patterns, and emotional reactions.

According to Bandura [12,13], there are four main factors that influence self-efficacy: (1) mastery experiences: individual’s prior experiences and performances, influenced by the success of previous experiences, which cumulatively increases their sense of self-efficacy; (2) vicarious experiences: the observation of activities performed by others, in which visualizing their accomplishments can increase their confidence in performing them as well; (3) verbal persuasion: individuals are encouraged to believe in their abilities through the use of suggestion, persuasion, or instruction; and (4) emotional arousal: emotions influence one’s performance, behavior, and perception of challenges, with positive emotions generally increasing confidence and vice-versa.

Self-efficacy is an important predictor of academic performance [3,14-19]. According to Rittmayer and Beier [20], individuals with high self-efficacy tend to set more challenging goals for themselves, envisioning a positive academic performance, thereby positively impacting STEM task performance and engagement. On the contrary, individuals who doubt their capacities (reflecting a low sense of self-efficacy) tend to avoid certain situations, believing that they are incapable of surpassing them and losing motivation [12,15,21], making them more likely to leave STEM education [22,23]. Self-efficacy may be a driving force in this process, helping shape career preferences [24] and aspiration [1,21,25-27].

Early exposure to STEM content in a child’s educational journey might be crucial for shaping STEM identity and self-efficacy [28-30]. Studies have proven the success of games in engaging students’ learning interest [24,31-35], with examples including how gamification has been applied in modern pedagogical strategies [32,36], and as a potential source for influencing self-efficacy [31]. Kinzie and Joseph [37] define a game “as an immersive, voluntary, and enjoyable activity (...) [that] provides a safe environment for taking chances and the opportunity to develop the knowledge and refine the skills required to succeed”. Using games in the classroom is becoming increasingly popular [31,33,34,38,39], facilitating learning through an enjoyable environment that fosters knowledge acquisition, learning achievements, and critical thinking, encouraging meaningful connections [31,33,34,40-42]. Reduced concern about failure, assumed within gameplay, contributes to a more positive learning experience [40,43], making games effective to introduce STEM-related scientific concepts [31].

#### Dynamics of Collaborative Activity

For contemporary challenges, collaboration has become a promising mode of human engagement [44-46], defined as “a continued and conjoined effort toward elaborating a ‘joint problem space’ of shared representations of the problem to be solved” [47]. It is also seen as an educational approach that improves learning outcomes [7,48-51] through group activities, such as information sharing and knowledge development between peers [48-50,52]. With a bottom-up approach, it leads to joint problem-solving [44,45,53] by combining game-based learning with student-led game design [54,55]. These joint interactions reduce frustration and increase confidence through peer support [56-58], enabling competence development by sharing personal experiences, knowledge, and ideas [59-62]. Developing games that support collaborative interactions can thus enable positive outcomes regarding STEM achievement, persistence, engagement, and problem-solving skills [7,50,56,63-65].

Recent studies [41,51,66-70] show a positive influence of game-based collaboration in self-efficacy: (1) Sung and Hwang [41], through pre- and postexperience questionnaires, explored collaborative game-based learning through knowledge organizing and sharing to increase the students’ interest; (2) Pan et al [69] found that digital game-based learning significantly improved students’ social collaboration, problem-solving skills, and motivation; and (3) Yin et al [70] studied the effects of pair programming in CT education, with collaborative learning significantly improving students’ self-efficacy compared to individual learning, where students with higher self-efficacy were more engaged in CT learning, enabling better CT performance and higher learning satisfaction.

Bardram [71] proposed 3 levels of dynamics that a collaborative activity entails:

Co-ordination: individuals carry out tasks to achieve the overall objective of the activity. At this lowest hierarchical level (the *“*how*”*), individuals are coordinated to perform the assigned functions/tasks, which are carried out individually, leading to a routinization of the work.

Co-operation: individuals interact to decide on a common object, still sharing the objective of the activity. In this intermediate hierarchical level, the objective is still stable, but task distributions are actively discussed and adjusted between each other depending on the object of work (the *“*what*”*).

Co-construction: individuals question the activity’s main objective. At this highest hierarchical level, reformulating the objective implies changes in the objects of work, tasks, and interactions. The overall objective or motivation is collectively reconstructed by the individuals (the *“*why*”*).

The hierarchy between the 3 levels is dynamic, reflecting transitions or breakdowns [71] between them upward and downward; we move upward when there is a reflection on the object of work (co-operation) or the entire objective of the activity (co-construction); or we move downward to implement the renewed objects from the new objective, implementing new tasks and routines.

#### CT and Its Components

CT is a problem-solving approach rooted in computer science, considered a vital contemporary skill and valuable in STEM education, as it addresses computational tools and problem-solving processes [72-75] for solving complex scenarios [76-78]. It involves conceptualizing problems in abstract ways, being taught to people of all ages [72-74,79,80], and expanded to various disciplines [81] (such as biology, geosciences, economics, and humanities [72]).

The CT approach is composed of four main characteristics [82-84]: (1) decomposition: “breaking down” the problem or task into smaller steps/parts; (2) abstraction: identifying critical information while ignoring irrelevant or unrelated details; (3) algorithms: developing a step-by-step solution or rules to solve the problem; and (4) pattern recognition: searching for similarities among and within problems, identifying patterns that enable information organization and help in problem-solving tasks.

CT is historically associated with programming skills [85], often approached through programming-oriented activities [86-91], resulting in a lack of valid measures that assess CT without using programming tools [84]. However, CT should not be reduced to formal coding, as it involves broader problem-solving processes, thinking at different levels of abstraction, and transferable competences across contexts [72,73,81,86,92] (including STEM education [75,81]). Games have also been identified as promising approaches to make CT more accessible and engaging [80,93], with game-based learning associated with active learning, motivation, engagement, and problem-solving, particularly in areas often perceived as difficult [94,95]. Playful engagement with CT, particularly in collaborative game-based contexts, may thus provide useful settings for supporting self-efficacy in CT learning [76,77,86], ultimately enhancing engagement in STEM fields.

### Research Problem and Objectives

We propose a collaborative game design, based on a low-resolution tabletop prototype through a playful probing approach [96], aiming to support self-efficacy in CT skills among children to promote interest in STEM. We introduce MeerPlay [97], a collaborative game centered on managing a colony of meerkats, where computational challenges are presented through a playful, collaborative approach, engaging players while enabling competence development of basic computational skills.

Considering this framing within STEM as a wicked problem [98], a transdisciplinary, long-term transformation that joins knowledge from multiple fields is needed to incite social change. To address this research gap, the presented paper-based prototype is part of a longer study that includes co-design sessions with children, which will result in a transmediation process to a mixed media game. In this first prototype, we explored the dynamics of collaborative processes [71] to conjecture how they may create opportunities for self-efficacy development.

The aim of this study was the design of a play environment that enables collecting evidence on how collaborative activities can contribute to the development of CT competences and support self-efficacy regarding them. We focus our investigation on answering the following question: What evidence can be found regarding the impact of collaborative activities in the development of self-efficacy regarding CT skills?

## Methods

### Overview

We adopted the dynamics of collaborative processes [71] to conjecture how a collaborative play activity could be designed to support self-efficacy development in CT skills. This conjecture led to the design and development of MeerPlay, exploring 3 collaboration levels while also welcoming and supporting dynamic transitions between these levels.

In this study, we sought to determine when and how a specific design influenced the development of self-efficacy; it is not enough to measure the change with a pre- and posttest approach. Our goal required uncovering evidence of collaborative gameplay events and how they support self-efficacy by observing, documenting, coding, and interpreting participants’ behaviors.

As such, our approach must diverge from existing literature focused on measuring self-efficacy [99-103], which relies mostly on pre- and postexperience questionnaires (verbal responses), adopting instead a content analysis method based on tracking and classifying the players’ behaviors during gameplay. This distinctive approach allowed us to interpret behaviors regarding both collaboration concepts and CT-related self-efficacy concepts, “since performance can not be faked” [104], while avoiding reliance on self-reported data. In addition, to ground our analysis on a deeper conceptual understanding of CT, we designed CT activities differently from traditional programming tools, focusing instead on CT as a set of problem-solving strategies and underlying components, as previously presented.

### Research Design

#### Overview

Our research design is structured as an iterative Design Science Research (DSR) approach, which is characterized by using “design as a research method or technique” [105], and is constituted by five phases: (1) awareness, where a new problem is perceived and characterized, raising interest to discover the “missing knowledge” [105]; (2) suggestion, where design ideas are formulated to explore the problem through a design proposal; (3) development, where a proof-of-concept is created to demonstrate the feasibility of the proposal; (4) evaluation, testing the designed artifact following a set of evaluation criteria, where gained information can justify returning to the awareness, suggestion, or development stages for adjustments and improvements; and (5) conclusion, as the end of a research cycle, synthesizing knowledge contribution, innovation value, and application in other scenarios.

In our research, we started by studying the current low levels of self-efficacy and motivation leading to STEM avoidance and dropout (awareness of problem), as well as design inspirations and concepts to gather insights into how cooperative behaviors could enable the empowerment of the participants during the gameplay activity, by developing competences that would increase their ability to perform in-game. A game design proposal was then formulated (suggestion) following a playful probing approach [96], focused on using play activities for data collection to investigate how participants engage with the artifact in a playable context. For that, a low-resolution paper prototype of the game was produced and used as a proof-of-concept of the playful probing approach (development) to perform gameplay rehearsals. The rehearsals were video-recorded, and individual semistructured interviews were conducted at the end of each session to gain further insights into the player’s perception and analyze modes of engagement (evaluation).

This study reports the evaluation of one iteration of the DSR process. It presents a cross-sectional study involving 5 playtesting sessions, analyzing data from a sampling population at a single point in time, aiming to find game design insights on the relationship between collaboration and CT-related self-efficacy. A qualitative study with the target audience was conducted (14 children aged from 6 to 11 years), with 5 gameplay rehearsal sessions and individual semistructured interviews, from which we sought evidence of how collaborative activities can support self-efficacy toward CT.

The qualitative content analysis of player behavior was based on an axial coding of categories related to meaningful events of competence development, collaboration, and self-efficacy sources. The axial coding was cross-checked and discussed among the 3 authors for consensus and consistent coding. We interpreted transitions in which players initially expressed difficulty but, through collaborative assistance, overcame them and performed new gameplay actions as a potential gain in self-efficacy, which we discuss as evidence of a relationship between these categories. In the end, we drew design insights for collaborative gameplay activities, supporting self-efficacy development in CT. The process is schematically explained in Multimedia Appendix 1.

#### Instrumentation: Design Proposal

We applied a low-resolution game prototype based on the work of Pinto et al [97,106], which proposed a cooperative design where players care for a meerkat colony, inspired by the collaborative social organization of this animal species. In this study, we provide an overview of the game, with a special focus on the design elements that support collaborative activities within the game.

Regarding the game’s theme, given children’s affinity with animals [107,108], and the intention of a collaborative environment, we sought species with a natural social organization and cooperative attitudes. Meerkats provided a suitable solution, as their cooperative social structure influenced both the game’s setting and the actions available for the players to perform. This led us to define 3 core functions [109,110], including territory exploration and development, defense against threats, and colony sustenance. As collaborative behavior was central to the design, the game was organized around a shared goal (the growth of the colony), which encouraged players to cooperate throughout gameplay.

The prototype was conceived as a collaborative environment for exploring CT through play, connecting its gameplay structure to STEM. Rather than introducing these competences through formal programming syntax, the game was designed so that children could engage with them implicitly by collaboratively defining the behavior of the meerkat entity through condition/action/result. In this way, the 4 CT dimensions (decomposition, abstraction, algorithmic thinking, and pattern recognition) were embedded in a playful and accessible activity [106].

The game design incorporated several activity modes [37,111] in a collaborative environment to promote mutual assistance in solving problems. Players participate in the game by defining the meerkat’s behaviors [106] as they move their characters on the board, roll the dice for action points, decide, and act to explore the space, gather resources, deal with dangerous encounters, and share action points with other players.

**Figure 1.**
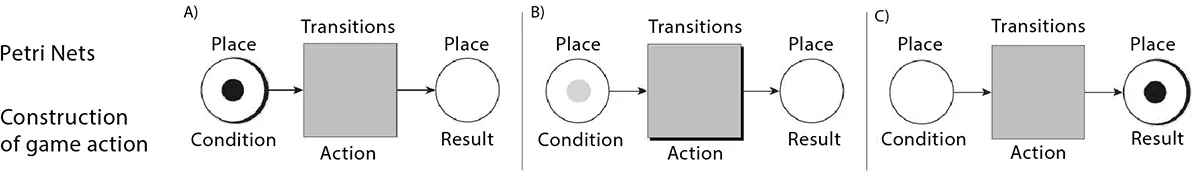
Adaptation of Petri nets to the construction of the player panel. Process: (A) Token in the place/condition; (B) Activation of the transition/action, change of the token; and (C) Token has moved through the transition to the following place/result. Adapted from Araújo and Roque [112].

In the game, players need to define the behaviors of meerkats by coding action sheets with preconditions, actions, and postconditions (or results). The construction of the meerkat actions is based on Petri-Net models in games [106,112] by associating their structure with the programming components for constructing the game characters’ actions (Figure 1). The process is carried out by defining conditions (Figure 1A) that, when verified, trigger or enable actions (Figure 1B) that produce results (Figure 1C), in the form of information or resources that can possibly satisfy other conditions.

The game’s actions operate when the preconditions are satisfied, either through the existence of the element in the game (inserted in the condition) or the direct contact of the meerkat with it (eg, the player finds the element hidden in the main board). This process triggers the action associated with this precondition, thus producing the player-specified result on the main board. Through this action-construction process, players practice competencies associated with CT skills [106].

**Figure 2.**
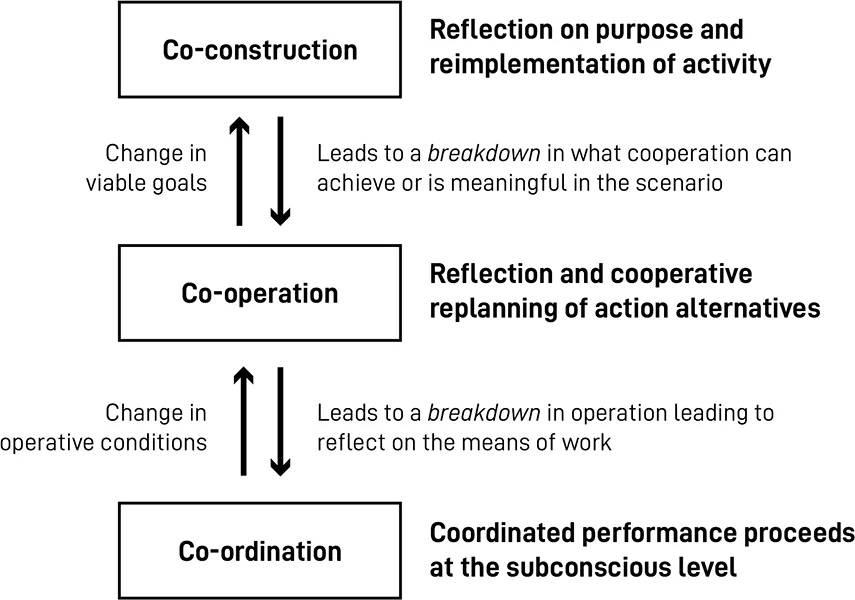
Dynamics of cooperative work, showing the relationship between co-construction, co-operation, and co-ordination, and the associated processes of reflection and stabilization regarding the object of work and the means of work (Adapted from Bardram [71]).

After defining the dynamics of the meerkat’s actions, the authors considered ways to design for collaborative gameplay with different levels of collaborative activity dynamics (Figure 2). For that, the 3 activity theory levels and their dynamics in collaborative activity [71] were considered.

Co-construction level: in order to facilitate social interaction and collaboration within the group, it is essential that its members share a common goal. For that, we proposed the shared purpose (the increase of the colony members).

Co-operation level: the game objective cannot be achieved alone, demanding interdependence among players. As such, the player’s panel for defining the meerkat behavior is shared, where players need to cooperate to discuss, distribute, and collaboratively define the actions/tasks that each meerkat will perform on the main board to achieve the overall objective of the collective activity.

Co-ordination level: task routinization and communication are important components for the game’s resolution, promoted by the game’s activities and the shared player panel. Players are required to communicate and agree on the definition of actions, coordinating the execution of these defined actions on the main board to achieve the game goal.

Each individual, after discussing and defining the player panels between all, will focus only on their function and the successful completion of their task on the main board. Considering that, we developed the following activities/tasks: (1) movement on the main board; (2) exploration of cells on the main board; (3) creation of burrows/tunnels on the main board; (4) collection of food on the main board; (5) unloading the meerkat food; (6) confronting predators/invaders/thieves on the main board; and (7) territorial control.

The main objective of players in this game is to grow the number of members in their colony. To accomplish this, co-operation is essential, as players must work together to define the meerkats’ behaviors, specifying them in each round and using the dice to take actions. Collaboration between players may manifest in the form of coordinating meerkat behavior coding, working toward shared goals, or sharing action points (dice values) between them. The game’s design is intended to encourage activities that promote communication and collaboration in order to develop effective strategies, learn how to code useful behaviors, and influence one another’s decisions to achieve common goals.

#### Instrumentation: Paper Prototyping for Rehearsal

**Figure 3.**
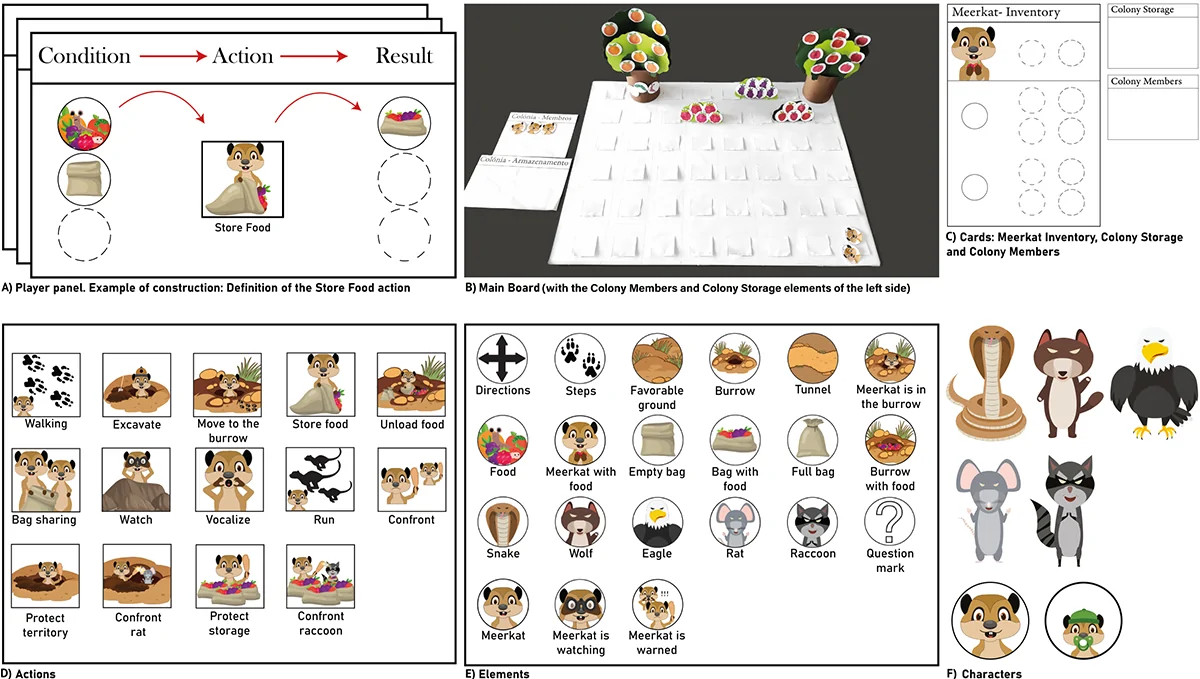
Paper prototype showing: (A) the player panel, with an example of the Store Food action; (B) the main board, including the colony members and colony storage cards; (C) the game cards, namely the meerkat inventory, colony storage, and colony members; (D) illustrations of the game actions used to construct the player panel; (E) illustrations of the elements used to construct the player panel, as well as those hidden in the cells of the main board for the activation of player panel conditions; and (F) illustrations of the game characters.

The game prototype consists of a player panel (Figure 3A) for coding the meerkats’ actions and a main board (Figure 3B), which defines the scenario where the meerkats enact the player’s choices.

The player panel was designed mimicking the Petri nets’ graphic structure [112]: it is formed by a set of cards, each with a condition area (with up to 3 possible conditions), one main action to be performed, and a result area (enabling up to 3 possible consequent results). This panel design gives the player freedom to set various conditions for the action to be enabled, combining different condition-action-outcome pairings to implement their strategy.

Considering these mechanics, we developed actions, based on the meerkat’s natural behaviors (Figure 3D), as well as elements to signify conditions or outcomes (Figure 3E). We also created visual components of the colony (Figure 3C), namely (1) a card with colony members, for keeping track of its size, (2) a card for storage, representing the players’ deposit of collected food, and (3) a meerkat inventory, to register the amount of food they carry, both in their hands and in the bag. Finally, enemies were also defined (Figure 3F).

The main board consisted of 2 overlapping A3 paper sheets, with the upper sheet divided into a 6×8 grid of cut cells to reveal hidden game elements. This design enables hiding resources and opponents on the main board, which are activated through actions defined in the player panel when discovered by the player. Trees and shrubs were also created to visualize food rewards, encouraging exploration.

Concerning game mechanics, players could use up to 8 behavior cards, which they collaboratively coded in each round to define the possible meerkats’ actions on the main board. Executing these actions depends on the discoveries made, as players explore the cells on the main board or by making appropriate changes in the game context (eg, to unload food requires excavating a burrow).

**Figure 4.**
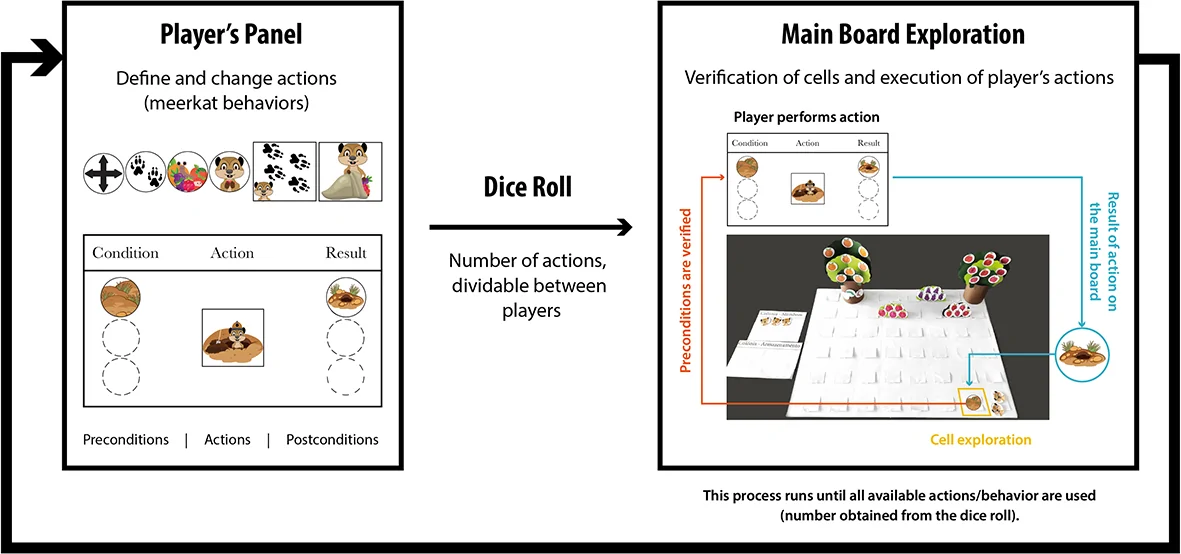
Gameplay loop, showing the construction of actions in the player panel following the dice roll, which determines the number of available actions, and the subsequent exploration of the main board through condition verification, action execution, and application of results. Main board exploration continues until all available actions/dice values have been used.

A round consists of three distinct phases (illustrated in Figure 4): (1) defining the actions on the player panel; (2) rolling the dice; and (3) the meerkat executing the actions (as defined on any players’ panels).

The game begins with 4 behavior cards on the player panel, 2 actions (walking and excavating), and 4 elements (directions, steps, favorable ground, and burrow). Additional elements and actions are unlocked gradually based on discoveries made during the game. Each player rolls a die, and the combined total determines the number of actions and movements the meerkats can perform in the round. Players can distribute these actions among themselves as desired.

The game concludes when all cells have been explored, all food (cells, trees, and bushes) has been collected, and all enemies are confronted on the main board.

### Study Participants

For this study, 5 gameplay rehearsal sessions (Table 1) were performed with a convenience sample accessed during postschool activities: 3 sessions composed of mixed-gender groups with 3 individuals: one group of 3 female individuals and a group of 2 female individuals. In total, the sample represents 14 individuals (9 female and 5 male), aged 6-11 years, and enrolled between the 1st and 5th grades.

This age range reflects the study’s early STEM exposure, enabling us to explore whether the game was appropriate for children of different ages within the target group. However, we assume this range might also be a possible limitation of the results, since the developmental differences between 6- and 11-year-old children are substantial. Even so, we could still identify, code, and find cases pointing toward openness to collaboration, indicating that data saturation had been reached.

**Table 1.** Sample categorization of the conducted 5-session study, presenting each players’ age and gender distribution.

Session	1			2			3		4			5		
Player	P1^a^	P2	P3	P4	P5	P6	P7	P8	P9	P10	P11	P12	P13	P14
Age	7	6	6	8	8	8	10	10	9	9	9	10	11	11
Sex	Male	Female	Female	Male	Male	Female	Female	Female	Female	Male	Male	Female	Female	Female

a P: player.

### Participant Recruitment

The recruitment process occurred within a school context, where the responsible teacher proposed the activity seeking volunteers. Following this process, we were able to recruit, alongside the teacher, the 14 participants described in the previous section, aiming to balance the age distribution across the gameplay sessions to ensure similar stages of cognitive development within each group. We also had substitute participants, if needed, when the first volunteers were not available.

### Ethical Considerations

**Figure 5.**
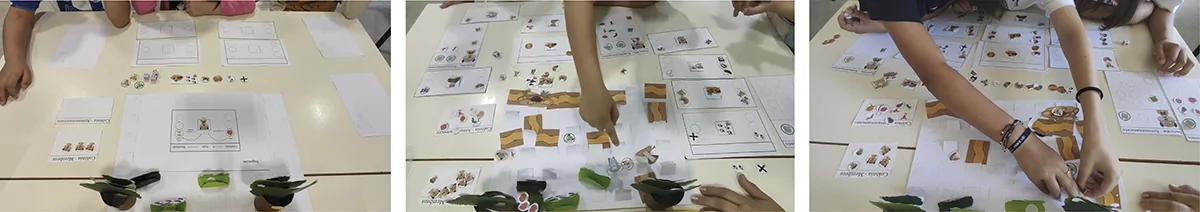
Photographs from the gameplay test session: (left) session beginning, showing the explanation of action construction using the player panel, through the standard example of the meerkat eating an apple; (middle) session midpoint, showing interaction with the rat invader, which takes over the tunnels; (right) session near-final stage, showing the continued exploration of the main board until all cells had been explored and food resources collected, including shrubs and trees.

As the study involved child participants, informed consent was obtained from all parents or legal guardians prior to participation. Consent covered participation in the study, video recording, interviews, and the use of collected data for research purposes. For publishing the results, we purposely selected images of the participants (specifically within the composition of Figure 5) that avoided any personally identifying features of the participants to fully protect their identity.

No financial or material compensation was provided. The study was approved by the Ethics Review Board of the Interdisciplinary Research Institute of the University of Coimbra (process 27_ID948). Privacy and confidentiality were safeguarded in accordance with the University of Coimbra’s Research Data Management guidelines and the General Data Protection Regulation (GDPR). All data were deidentified for analysis, with participants referred to in the study by anonymized codes (P1-P14).

### Data Collection

Five gameplay sessions were performed, ranging from 1 hour 50 minutes to 2 hours 38 minutes, resulting in a total of 10 hours and 45 minutes of audiovisual recordings. Explicit permission was obtained from the children’s parents/guardians for the interviews and the recordings.

Each session began with the researcher presenting the game’s theme and components, the dynamics and rules on the main board, and the action definition of the player panel, with an example of a condition-action-result construction. During gameplay, doubts and problems were clarified with the least possible interference of the researcher, ending with the individual semistructured interviews.

Each session was audiovisually recorded, using an overhead tabletop camera and microphone, focused on the board and pieces to avoid facial identification (Figure 5). Voice dialogues were later transcribed. Observations of recorded players’ behaviors and meaningful game events were subject to qualitative content analysis, as described in our qualitative content analysis subsection.

We sought to allow participants (children) to describe their experiences more freely, to articulate their experience in their own words, and to provide richer accounts of their perceptions during gameplay. With this purpose, we adapted the guide by Bandura [113] to create a postgameplay semistructured interview, intentionally designed as open-ended, addressing aspects of the gameplay experience and how collaboration influenced underlying perceptions of self-efficacy support during gameplay.

Given the age of the participants, this was considered more suitable than requiring them to quantify their experience through scaled responses, as “closed-ended questions can lead to ambiguous, incomplete, and/or inaccurate responses from children, particularly when compared to open-ended questions” [114]. As Bandura [113] stated “there is no all-purpose measure of perceived self-efficacy”, which should be assessed depending on each research context and situational demands.

### Qualitative Content Analysis

The content analysis was conducted by the 3 authors (VMP, MS, and LR), and consisted of four steps: (1) video observation and memoing (to gain awareness to the content to be classified, duration, frequency, and separation), (2) selection of evidence units to be coded along the categories relevant to the study goals, (3) data coding related to behavioral evidence of CT competences [106], collaborative activity and self-efficacy events, and (4) interpreting the interviews to support the classification of behaviors (data triangulation).

### Coding Scheme

Regarding the coding of the category of competence, 3 subcategories were identified in data, including reading and interpretation; condition-action-result; and action chaining. The process of associating this competence with CT was retrieved from Pinto et al [106], and can be further consulted there. In Table 2, we expose the coding subcategories along with their definitions and examples of evidence collected.

**Table 2.** Coding of competence development subcategories (reading and interpretation, condition-action-result, and action chaining) associated with the computational thinking’s (CT’s) skill set (abstraction, algorithms, decomposition, and pattern recognition) with the corresponding definitions and illustrative evidence.

Subcategory and description	Coding frequency	Illustrative evidence
Reading and interpretation: ability to read and understand the construction carried out in the player panel. Associated with the abstraction in CT^a^, which involves the ability to focus on essential aspects of the representation. In the game, players must comprehend the symbols to encode information and understand game mechanics and instructions. By abstracting important information, players can understand how the relationship between game elements enables them to make decisions and elaborate actions.	Session 1: 157 Session 2: 57 Session 3: 72 Session 4: 151 Session 5: 151	“If we find good terrain, we dig and make a tunnel”. “That makes sense.” “He has food (points to the food element), then he will store it (points to the store action), and he will eat (points to the meerkat with food).” “We can dig a tunnel because we have a burrow. We need good ground to excavate and make a tunnel, or if we have a burrow, we can excavate and build a tunnel...”. “We can excavate a tunnel because we have a burrow”. The player suggests that they build a tunnel in the cell adjacent to the burrow, creating a connection with other tunnels.
Condition-action-result: construction of the player panel. Identification of the need for certain elements to trigger the desired action to obtain a determined result. Associated with the algorithmic competence of CT. In the game context, players create algorithms by selecting and organizing the elements in the action sheets by defining the preconditions, actions, and postconditions in the player panel.	Session 1: 467 Session 2: 397 Session 3: 290 Session 4: 499 Session 5: 543	Action construction: “We have to pick up here (steps element)... I put it here (inserts the steps element into the condition area of the player panel)... I put it here (inserts the walking action into the action area of the player panel) and this... (inserts the directions element into the result area of the walking action) here!”
Action chaining: the need for developing an action to create conditions for another action. This subcategory helps to identify the chaining logic and is associated with the decomposition competence of CT. In the game, it is represented as the ability to break down the activity into steps, where players plan how to arrange actions in a specific order, considering the dependencies, to achieve the activity outcome.	Session 1: 58 Session 2: 48 Session 3: 41 Session 4: 95 Session 5: 46	“The meerkats are watching, and then they will vocalize.” “To store, we need food, a meerkat, and a place for us to store (referring to the unload action), that is.”

a CT: computational thinking.

Pattern recognition is a critical aspect of CT, involving the ability to identify recurring patterns, elements, or sequences of actions. Notably, it emerges as an essential competency intertwined with all subcategories of competences (Frequencies: Session 1: 6, Session 2: 7, Session 3: 2, Session 4: 7, Session 5: 6). This association across multiple subcategories underscores its comprehensive nature, as it enables players to identify symbol patterns, sequence actions, and forecast outcomes, thereby expanding their problem-solving skills and overall gameplay experience.

For the collaboration category, 3 subcategories were considered [71], including co-construction, co-operation, and co-ordination. In Table 3, we find the coding subcategories along with definitions and examples of illustrative evidence collected.

Concerning the coding of the category of self-efficacy*,* 4 subcategories enabling behavioral changes were used [12,13], including mastery experiences, vicarious experiences, verbal persuasion, and emotional arousal. In Table 4, we expose the coding subcategories along with their definitions and examples of evidence collected.

**Table 3.** Coding of the subcategories of collaboration (co-construction, co-operation, and co-ordination), with corresponding definitions and illustrative evidence.

Subcategory and description	Coding frequency	Illustrative evidence
Co-construction: situations where there was an attempt to reformulate, question, or negotiate the aim or conclusion criteria for the shared play activity or strategy to pursue within the game.	Session 1: 2 Session 2: 2 Session 3: 0 Session 4: 9 Session 5: 0	Interaction among players: P11: “When does this end?” P9: “There is an apple! The one who catches the apple wins the game!” P11: “Oh, so the one that catches the apple wins the game, is that right?” P5: “When will it finish?” P4: “Do we have to pick up all the food from the trees, is that it?”
Collaborative co-operation: scenarios that involve discussing and sharing strategies for achieving the game objective, specifically focusing on the tasks to be performed and determining which actions would be beneficial and necessary for the game’s development.	Session 1: 393 Session 2: 422 Session 3: 227 Session 4: 1244 Session 5: 682	Interaction among players (conversation about walking action): P12: “So wait. We need a meerkat.” P14: “And directions”. “Exactly... And now, what do we need?” P12: “In the action of walking, we can reach a tree to get fruits”. “In other words... We need a meerkat, directions, and to walk.” P14: “Walk.” P12: “That’s it”. “My people, many of us need steps. How are we going to walk without directions?” P14: “Exactly.” P13: “Yeah... Without steps…” laughs. P14: Action construction: Inserts the steps element into the condition area of walking action: “We need steps to walk.” P13: “To walk.” P12: “Of course, how else are you going to walk? On a cart, right?” laughs.
Co-ordination**:** performing the assigned actions or tasks without questioning or doubt to achieve the game’s objective	Session 1: 270 Session 2: 319 Session 3: 312 Session 4: 373 Session 5: 280	P13: moves 3 cells. P14: assists in P13’s movement, keeping track of the cells for the movement. P5: builds a tunnel. P4: assists in counting the actions used by P5.

**Table 4.** Coding of the subcategories of self-efficacy (mastery experiences, vicarious experiences, verbal persuasion, and emotional arousal), with corresponding definitions and illustrative evidence.

Subcategory and description	Coding frequency	Illustrative evidence
Mastery experiences: execution/performance of activities	Session 1: 520 Session 2: 409 Session 3: 374 Session 4: 729 Session 5: 618	Performance sequence of P5: Players discuss which activities to perform during the game. P5 suggests constructing a burrow on the main board: “Make the burrow”. The player proceeds to the construction of the excavate action on the player panel by inserting the burrow element into the condition area. Upon analyzing the construction, P5 realizes their own mistake in placing the burrow element in the condition area, as the intention is to excavate a burrow. P5 promptly removes the burrow element from the condition area and performs the correct construction.
Vicarious experiences: observation of others’ performance, influencing the individual’s performance	Session 1: 15 Session 2: 7 Session 3: 11 Session 4: 13 Session 5: 6	Interaction among players: P8 suggests that P7 inserts the confront raccoon action into the player panel, “Put it in (points to the confront raccoon action)”, “Put it in the middle (referring to the action area on the player’s panel).” P7 action construction: inserts the confront raccoon action. P8 action construction: “And then you put this one”, referring to the raccoon element into the condition area, “and then it’s there (referring to the question mark in the result area).” P7 action construction: insert the raccoon element into the condition area of the confront raccoon action and insert the question mark into the result area.
Verbal persuasion: sharing of ideas, persuasion, and encouragement	Session 1: 29 Session 2: 60 Session 3: 17 Session 4: 176 Session 5: 79	Interaction among players: P3 suggests that player P2 stores the cell’s food, “Grab the food... Grab the food... Grab the food…” P2 moves. P3 reacts to P2’s movement, “Grab the food... you didn't grab the food.” Interaction among players: P5 reacts to the information of food loss, “NOOO! P4, grab it”. Suggests that P4 go store the food in P6’s cell, as he is the only one with an empty inventory. P4, “Hold on, I’ll grab it” (laughs). “If I can, I'll make a tunnel”. P5 grabs P4 and shakes him while saying, “P4, please, grab it”. P4 performs the store of the cells’ food.
Emotional arousal: expression of emotions and feelings	Session 1: 179 Session 2: 231 Session 3: 78 Session 4: 704 Session 5: 75	Interaction among players: P4 reacts to victory, “Woohoo!” P6 screams, “Aaaaaiiiiiiiiiiiiiii.” P5 “GOAL, I did it!” P4 “The raccoon lost, it even flew.”

### Content Analysis Procedure

*“*Where quantitative content analysis is helpful in answering ‘what’ questions, qualitative content analysis can be helpful in answering ‘why’ questions and analyzing perceptions*”* [115,116], which is exactly the methodological perspective that we followed for retrieving behavioral evidence between collaborative activity and self-efficacy support. Axial coding followed a deductive process, using the categories and subcategories based on foundational theoretical lenses, from the presented literature, namely: (1) the 3 modes or layers of collaborative activity [71], (2) the known predictive factors from self-efficacy [12], and (3) the 4 CT skills [82-84,106].

For this analysis, although we recognize Bandura’s guide [113] focuses on measuring quantitative variations through item scores, we needed to track the relations between aspects of the gameplay activity as sources of self-efficacy by choosing to code for CT and collaboration events. Instead of scoring the items quantitatively, we observed specific participants’ behaviors during the gameplay experience and coded behavioral evidence into qualitative expressions corresponding to defined levels of collaborative activity, sources of self-efficacy, and CT competences. Relevant data were thus retrieved from both the video recordings and the interviews (based on the same coding scheme), with the postvideo interviews used to disambiguate the classification of behaviors (data triangulation). The questions used in the interviews are available in Multimedia Appendix 2.

Our axial coding process was conducted through the following steps:

1. Unit of analysis: we split the videos into 30-second segments for coding evidence by the addressed subcategories, including the participants’ behavior and vocal expressions. This interval was selected according to the required level of detail of recognizable actions for the phenomena under analysis. As Pesch and Lumeng [117] state, “the time interval for coding must be carefully selected” and “depends on the level of detail that the researcher wishes to capture”. Likewise, Jewitt [118] argues that researchers should “decide on the scale you will look at and how much data you need to address your question”, as video analysis is time-intensive and therefore “can tend to focus on short segments of video data at a micro-analytical level”. Heath et al [119] also highlights the analytic richness of brief video fragments, recommending short fragments, including those up to 30 seconds.
2. Initial coding: the authors discussed the inclusion/exclusion conditions, supported by the interview transcripts and observational notes, during the first coding round. Initial coding revealed an interrater reliability of 96%, which motivated the discussion and agreement by the 3 authors (VMP, MS, and LR) [115,120] of evidence that generated doubts or divergence. Initial coding of empirical evidence confirmed that the 30-second interval allowed a good balance between analytic depth, the amount of evidence captured, and coding feasibility. It enabled sufficient interactional context for interpreting the coded evidence, maintaining a manageable and consistent coding process across the data.
3. Merge and review: all the data gathered from the 5 sessions were merged into a single document, resulting in 5653 lines of coded evidence. After this merge, all subcategories were reviewed and any content units flagged as still in doubt were correctly coded by all authors to ensure consistency.
4. Process-oriented contextual analysis: in the fourth stage, we analyzed possible associations between the categories, identifying trends and patterns of activity signaling the skill evolution of individuals. This stage involved a process-oriented reading of found evidence, through which some influences became clearer when interpreted in the context of action sequences rather than as isolated coded units. This process-oriented interpretation also relied on the interview transcripts to check with the participants’ own perspectives.
5. Achieving saturation: due to the fact that we worked in a deductive form using categories previously defined for the analysis, it was not practical to run iterative data collection until no new themes appeared. We collected data from the gameplay cases that were available to us and processed them according to what is usually considered good practice in qualitative studies. Data saturation was noticeable to the extent that certain observable behavior patterns did emerge, as would be expected from an experience that is partially configured through the game design and partly autonomously interpreted by players. For our study goals, saturation is also a relative concern, since we are aiming to identify design insights on collaborative gameplay that constitute opportunities to foster self-efficacy, which are independent of frequency and can be identified from classifying a single occurrence.

### Methodological Integrity

The sample data were collected in a school context, providing a set of participants with diverse ages and sexes (within the target group) for our gameplay sessions. From them, we could analyze evidence to answer our main research question and goals, regarding the impact of collaboration on the development of self-efficacy in CT skills.

The responsible researchers’ perspectives were conveniently managed during the gameplay sessions, with the least possible interference from her. Within the coding analysis, the initial coding showed an interrater reliability of 96%, with the remaining discrepant coding being discussed and resolved among the 3 authors.

All findings are grounded in analyzed evidence, following a qualitative content analysis consisting of (1) video observation and memoing, (2) selection of evidence to be coded, (3) data coding, and (4) interview interpretation, which were used to support behavior evidence and enable data triangulation (schematized in Multimedia Appendix 1). The findings were properly compared to existing literature, describing both the similarities and contributions that our study provides (described in the Discussion). The results meet the main need and research question to find evidence on how collaboration encourages discussion, peer support, and competence development, thereby supporting self-efficacy in CT.

## Results

### Overview

In this section, we present the connections found between events of collaborative activity and events signaling support for self-efficacy in CT. We first present a summary of the evidence frequency found. Then we present specific instances when players exhibited some form of skill acquisition, transitioning from a situation when they expressed difficulty to another when, through collaborative activity, they were able to perform new gameplay actions. We interpreted these transitions as an influence in self-efficacy that could translate a relationship between these categories.

### Coding Frequency

Regarding the CT dimension, condition-action-result was the most frequently coded subcategory across all 5 sessions (467; 397; 290; 499; and 543). This may be related to the central role of the player panel in the gameplay activity, as players were repeatedly required to define preconditions, actions, and results in order to construct entity behaviors. Reading and interpretation was also identified across all sessions (157; 57; 72; 151; and 151), while action chaining appeared less frequently (58; 48; 41; 95; and 46), but remained present throughout the collected data. Pattern recognition was observed across sessions with lower frequencies (6; 7; 2; 7; and 6), possibly because it was less easily identifiable in isolated coded units, depending more on the analysis of related sets of evidence. Overall, it appeared to function mainly as a supporting competence associated with the other CT-related subcategories.

In the collaboration dimension, co-operation was the most recurrent subcategory across all 5 sessions (393; 422; 227; 1244; and 682), indicating that players frequently discussed tasks, shared strategies, and negotiated actions to progress through the game. Co-ordination was also consistently observed throughout the collected data (270; 319; 312; 373; and 280), reflecting the operational execution of agreed actions and mutual support during task performance. Co-construction appeared less frequently (2; 2; 0; 9; and 0), which may be related to its higher-level and usually the rarest role within collaborative activity, as it involved moments in which players questioned, reformulated, or negotiated the aim or conclusion criteria for the shared play activity or strategy to pursue within the game. In Session 3, particularly, co-operation appeared less frequently than co-ordination, possibly because the 2-player group seemed to discuss/define actions more efficiently and move more directly to task execution and automating/routinizing behaviors.

Regarding the self-efficacy dimension, mastery experiences were the most recurrent subcategory across all 5 sessions (520; 409; 374; 729; and 618), which is consistent with the performance-oriented nature of the gameplay activity. Emotional arousal was also frequently observed, particularly in moments of success, excitement, or tension during gameplay. Although verbal persuasion appeared with lower frequencies (29; 60; 17; 176; and 79) than mastery experiences and emotional arousal (179; 231; 78; 704; and 75), it was still notably present, especially in Session 4, suggesting that its occurrence may also have depended on the communicative dynamics and willingness of each group to verbally support one another during play. Vicarious experiences were the least frequently coded subcategory (15; 7; 11; 13; and 6), which may seem unexpected in a collaborative game context. A possible explanation is that the coding of isolated evidence units did not always allow this type of influence to be readily identified, as vicarious experience often required the analysis of related sets of evidence, in order to infer how the observation of another player’s behavior influenced subsequent performance. Accordingly, the illustrative cases can clarify the role of vicarious experience, as this influence became more visible through a process-oriented reading of sequences of related evidence and through the participants’ interviews.

### Illustrative Cases

In these gameplay rehearsals, the collaborative support among players encouraged discussion on how to construct actions, including clarification and explanation to other players. This environment may have encouraged players to express their doubts and instruct one another on how to proceed in certain gameplay situations, identified as instances of verbal persuasion [12,13]. The players also had the chance to observe and learn from the behaviors of other players during the game, which created opportunities for vicarious experiences, which are predicted [12,13] to increase the players’ confidence and encourage them to try to perform the task for themselves, consistent with mastery experience [12,13].

#### Cooperative Gameplay Levels or Dynamics

Analyzing the set of evidence, we could observe how participants communicated and collaborated to overcome challenges and achieve the game’s goal of increasing the colony. Players engaged in a cooperative dialogue to determine the most beneficial actions and how to execute them (co-operation level [71]). This process allowed errors to be identified, which encouraged mutual assistance and reflection on how to solve them. This created multiple opportunities for supporting mastery experiences through verbal persuasion and vicarious experiences [12,13], influencing the learning process and confidence in subsequent activities. Interactions at this stage through the mutual support enabled the accomplishment of the game’s goal, leading to players sharing their available actions (represented by the dice roll each round) and eventually coordinating their tasks (co-ordination level [71]). In this illustrative case (Session 1), we can observe the influence of collaborative dialogue on the action construction in the player panel:

- P2 incorrectly inserted the tunnel element into the result area of the walk action. Through collaboration among players to identify what would be necessary to perform the excavation, and with P1 demonstrating the correct place to insert the element (vicarious experience [12,13]), P2 was able to recognize and correct the mistake (impact of vicarious experience, resulting in performance: mastery experience [12,13]).
- P3, also by pointing to the element in the condition area, demonstrated what would be necessary to perform the action, completing the construction. This demonstration (vicarious experience [12,13]) influenced the other players, allowing the correct verbalization of the action construction, promoting the consolidation of the game’s skill reading and interpretation (mastery experience [12,13]), and thus assisting in the foundation of the dynamics of the competence condition-action-result (Textbox 1).

Textbox 1. **Interaction regarding excavation action.**

Interaction among players, conversation about excavation action:

P2 action construction: insert the tunnel element into the result area of the walking action.

Dialogue about tunnels:

P2: *With a shovel.*

P3: *Excavate*.

P2: *A shovel for excavating.*

P1 indicates the place where the element needs to be associated, pointing to the excavate action on the player panel.

P2 action construction: remove the tunnel element from the result area of the walking action and insert it into the result area of the excavate action.

P1 says that they need a shovel to excavate: *With a shovel*.

P3 reacts to the affirmation of P1, showing that what they really need is good ground, pointing to the favorable ground element in the condition area.

P1, through the demonstration of P3, initiated the reading of the construction: *Favourable ground…*

P2: *We want to make*…

P2: *A burrow!*

#### Opportunities for Self-Efficacy

Later in the game, P2 encountered a more complex scenario than the one previously presented, when a series of events are meaningful as sources of self-efficacy:

- P1 provided clarification on the appropriate action regarding the empty bag element (verbal persuasion [12,13]).
- However, despite this guidance, P2 once again inserted the element in the incorrect location. The distinguishing factor this time was that P2 demonstrated their own analysis of their construction, leading them to identify and rectify their errors independently (mastery experience gained [12,13]).
- When the researcher questioned P2 about the outcome of the constructed action, P2 showed an improvement in interpreting and applying game-related competences. Additionally, P2 was able to articulate the conclusion he had reached, demonstrating his comprehension of the adjustments. P2 demonstrated resilience, despite occasional mistakes and struggles in identifying the correct areas for constructing actions on the player panel. Collaborative interactions and observation of others' actions helped P2 recognize and rectify errors, thereby improving their performance. This perseverance can signal a positive result from the collaborative environment, where peer support is constantly available for problem-solving. This supportive atmosphere enabled P2 to feel more comfortable in making mistakes, enabling them to improve their construction skills throughout the game (Textbox 2).

Textbox 2. **Interaction regarding storing food in the bag.**

P1 states that to store the food in the bag they need an: *Empty Bag*.

P2 action construction: inserts the empty bag element into the unload action condition area.

P2 states the benefits of the bag: *I’ll fill it with 4 fruits.*

She identifies the action construction mistake and removes the empty bag element from the condition area of unload action and inserts it into the store action condition area. She reads the construction: *Empty bag…4 fruits.*

Research: *And what will this change result in?* and, pointing to the store action result area, says the meerkat here is storing the food in its hand (referring to the meerkat element with food).

P2: *I know, unload*… referring to what happens after having 4 fruits in the bag. *...from the bag.*

Action construction: inserts the bag with food element into the store action result area, stating, *Bag with food. I understand now... I have an empty bag, I'll have to put another 4 fruits here* (referring to the inventory)*.*

#### Emergence of CT

While engaging in cooperative gameplay, we observed players discussing and clarifying their thinking about how to define creature behaviors to better address challenges posed in the game environment. The following evidence (Session 5) exemplifies this clarification regarding the dynamics of the condition-action-result (algorithms: CT [82-84]), highlighting the importance of dependencies in the condition area through verbal persuasion [12,13]:

- Players analyzed how they should construct the confront action, considering the new predator (snake).
- P12 decided to remove all the elements they had in the condition area of the confront action.
- P14 alerted the players to the need for elements in the condition area for the activation of the action, emphasizing the condition-action-result dynamics (Textbox 3).

Textbox 3. **Interaction regarding the confrontation action.**

P12 verbalizes what to do regarding the snake, *Confront*.

P13 agrees with confronting, *Confront*.

P12 analyzes the construction of the confrontation action.

P14 alerts that there are more actions associated with predators (preventive).

P12 verbalizes that they need to remove the wolf element of the condition area of confront action.

Action construction: remove all elements from the condition area of the confrontation action.

P14 alerts that they need elements in the condition area, *Eeeee, but we need a condition for an action*.

P12 and P13 state: *Yes*.

#### From Collective to Individual Self-Efficacy

A scenario from Session 3 demonstrates an illustrative case on how the collaborative activity created opportunities for self-efficacy. This influence leads to vicarious experiences and verbal persuasion, resulting in the mastery experience of the player [12,13]. In the following sequence of evidence, collaboration between players is observed in defining the task through discussion and signaling of elements for construction:

- Players deliberated on the enemy’s behavior and collectively decided on the performance of the task (co-operation level [71]).
- Player P8 assisted P7 in constructing the action by identifying which elements were needed and their respective areas in the player panel. This collaboration promoted the demonstration (vicarious experience [12,13]) by P8 of how the action construction should be executed.
- Simultaneously, P8 encouraged (verbal persuasion [12,13]) P7 to carry out this process. With encouragement and support from the other player, P7 executed the action construction (mastery experience [12,13]) with confidence, potentially influencing their sense of self-efficacy during the game activities (Textbox 4).

Textbox 4. **Interaction regarding the confrontation and protection actions.**

P7 reacts to the information about the raccoon stealing food, *Then we have to protect.*

P8: *Then we have to use this* (points to the confront raccoon action).

P7: *What if we didn't have food?*

P8: *Would it steal a lot of food from us?*

P7: *But we could have just finished storing in the burrow*, referring to unloading food into the burrow.

P8 encourages P7 to insert the confront raccoon action on the panel, *Put it in* (points to the confront raccoon action)… *Put it in the middle* (referring to the action area on the player panel).

P7 action construction: inserts the confront raccoon action.

P8 dialogue about the action: *And then you put this…* referring to the raccoon element into the condition area, *and then it’s there* (referring to the question mark into the result area).

P7 Action construction: insert the raccoon and question mark in the respective place.

#### Redefining the Activity

A last illustration from Session 2 demonstrates how the game challenged players to modify the initial rules (co-construction level [71]), fostering collaboration within the group:

- Following a discussion about the challenges of an individual confronting a predator, P4 proposed a behavioral change: the collective relocation of all meerkats (players) to the confrontation zone to assist their teammates in achieving success in the game’s goal of preserving the colony. This collaborative decision-making process not only showcased teamwork but also positively supported the players’ self-efficacy, enhancing their confidence to perform the game challenges, as they knew they would have collective support (Textbox 5).

Textbox 5. **Changing the game’s rules.**

P4 suggests a solution to help player P6 in the confrontation against the eagle, suggesting that all players come together to assist in the confrontation: *She calls us, and we get there and pow pow pow* (makes punch gestures with his hand).

P5 and P4 move to the edge of P6’s meerkat and perform, collaboratively, the same activity as P6.

#### Cooperative and Competitive Dynamics

Overall, collaborative behavior was observed across all gameplay rehearsals. However, in 2 groups, collaboration coexisted with competitive dynamics, which appeared to take different forms depending on the participants’ age. In Session 4, involving 9-year-old participants, competition took a more playful and friendly form and did not appear to affect collaboration. Rather, participants challenged one another while still working toward collaborative goals, with the idea that whoever completed the task first would be the “winner”. Gameplay was therefore characterized by social interaction that supported collaboration while also fostering beneficial competition. Playful attitudes, such as teasing and joking (emotional arousal [12,13]), reinforced both collaboration and competition. This balance created a dynamic gameplay experience, in which participants supported each other while also seeking individual recognition, blending mutual assistance with personal achievement. By contrast, in Session 1, involving participants aged 6-7 years, the competitive dynamic was less playful, observing a stronger sense of ownership over individual resources, which in some situations led to conflict. However, these difficulties were related less to the collaborative nature of the activity itself than to the sharing of resources (perceived as a personal decision). When the task focused on constructing actions on the player panel, players collaborated by supporting each other with guidance on what to do. They appeared willing to collaborate through ideas, encouragement, and procedural support, while showing greater reluctance to share actions and dice values. This became particularly evident when players were asked to share the value obtained from a dice roll, or when the dice was exchanged between them. Although all the dice used in the game were identical, P3 claimed that P2 had taken her dice and attempted to retrieve it from P2’s hands, which resulted in a conflict between the players, with P3 even beginning to cry. Textboxes 6-8 illustrate these interactions.

Textbox 6. **Sharing dice values.**

- P2 expressed a wish to share the values: *Share... Yes, share it with everyone.*
- P3 stated that they did not wish to share the values: *No.*
- P2 reaffirmed a willingness to share: *Yes.*
- The researcher reminded the players that they had to reach an agreement amongst themselves regarding how to divide the values.
- P1 stated: *Each one keeps their own value.*

Textbox 7. **Negotiating dice values.**

In another situation, in which a player attempted to negotiate a dice value:

- P2 asked P3 for an action/dice value: *Will you give me one?*
- P3 stated that they did not wish to share their action with P2: *No.*

Conflict then emerged around the physical representation of the dice:

- P3 complained that P2 had taken their dice: *Oh P2, you took it! That is my dice.*
- P2 took another dice from the table and offered it to P3.
- P3 responded: *Damn! That was my dice.*

Textbox 8. **Conflict escalation regarding the dice.**

The escalation of the conflict regarding the dice:

- P2 had allegedly taken P3’s dice.
- P3 complained about P2’s behavior and attempted to retrieve the supposed dice from P2’s hands. Unable to do so, P3 took the dice that was on the table instead.
- P3 then began to cry, stating that P2 had scratched and hurt their hand.

#### Age and Reception

The previous example was the main situation in which age appeared to have influenced collaboration dynamics, particularly regarding difficulties in sharing objects or resources perceived as their own. However, the limited sample size does not allow stronger conclusions to be drawn regarding age-related effects, and no other meaningful event regarding age difference was observed beyond this case. Despite this conflict, players’ feedback remained positive, suggesting that these tensions did not affect their overall perception of the game. All players stated that they liked the game and considered it to have been designed for them, praising its fun nature, the challenges involved, and the fact that it was played together with others, which is especially relevant, given that they consistently expressed a preference for collaborative play. Overall, these findings suggest that the game was well received across the sample’s age range represented. This positive reception was further reflected in the interviews concerning collaboration and self-efficacy.

During interviews, players’ perceptions aligned with observations made during the sessions, indicating that collaboration was an important problem-solving factor that influenced their in-game behavior. When asked if observing another player helped them resolve game challenges (vicarious experience), all players (n=14) responded positively. Eight players mentioned that witnessing a fellow player’s actions inspired them to emulate those actions, with comments such as “Seeing others playing and building actions helped me understand how I could also do it” and “I saw my colleagues playing, and over time I tried to do the same”, further illustrating how observing others’ gameplay influenced their own strategies and actions.

Additionally, players mentioned that collaborative dialogues also played a significant role. Five players highlighted verbal suggestions and incentives during gameplay (verbal persuasion), with comments such as, “Yes, because she spoke with me” and “Yes, because that is a group game where everyone helps. They encouraged it”. The interviews highlight the potential impact of collaboration in supporting different forms of self-efficacy, facilitating effective gameplay and problem-solving.

## Discussion

### Overview

Research has indicated that collaboration can improve learning outcomes [7,48-51], and has been associated with positive outcomes in STEM achievement and persistence. From the analyzed gameplay rehearsals, by applying a qualitative content analysis, we found recurrent evidence regarding the benefits of collaboration for competency development and self-efficacy, particularly in CT activities [12,13,71,82-84]. We observed player interactions that revealed how collaboration facilitated error identification, mutual assistance, and dialogical reflection for their correction, ultimately enhancing in-game problem-solving skills and the perceived individuals’ confidence in their abilities.

### Principal Findings

Our findings show that collaborative interactions were key moments leading to problem-solving, which consequently contributed to improved individual performance. Players engaged in discussions to understand the condition-action-result (algorithms: CT [82-84]) dynamics, fostering not only problem-solving but also a shared understanding of game challenges and strategies. Social interactions promoted task discussion and modification, sharing goals, and distributing activities between them. Players engaged in collaborative decision-making to achieve the game’s ultimate goal. This involved engaging in cooperative dialogue [71] to determine the most beneficial actions and their effective execution, leading to task completion through shared actions and coordinated action [71] execution.

By playing in a team, participants learned from each other’s strengths and experiences, promptly assisting each other (verbal persuasion and vicarious experience [12,13]), resulting in a deeper understanding of the game activities, consistent with in-game progression, and fostering a complex, mutually supportive, challenging, and encouraging environment that led to several events coded as self-efficacy sources [12,13]. The overall supportive nature of collaboration, coupled with a playful attitude, created a motivating and engaging environment conducive to learning and flexible problem-solving, necessary for experimenting and achieving mastery experiences [12,13]. Furthermore, the designed form of collaborative gameplay exposed the players to others’ perspectives and resolutions, creating vicarious experiences [12,13] from which players could expand their skill set and seemingly increase confidence in handling various situations.

These findings align with the existing body of knowledge that emphasizes the positive effects of collaborative learning, particularly in programming education [56,59-61,121]. As Sung and Hwang [41] state, traditional science education frequently fails to engage and encourage students, for which collaborative game-based approaches should be explored to increase students’ engagement. Through the peer support that emerges from these joint exchanges, confidence is increased, frustration reduced, and support is given to the development of competence [56-58,122]. Collaborative activities may promote a discussion environment where each member contributes their experiences, knowledge, and perspectives, leading to improved learning performance [60,61] and self-efficacy support when compared to individual learning contexts [70], and greater satisfaction among individuals [56-58].

With our methodological approach, we differed from previous studies (focused on measuring aggregate results in self-efficacy [99-103] through pre- and postexperience questionnaires) by intending to study the design elements and analyze behaviors that influenced these collaborations. Our findings suggest the Meerplay game design and developed prototype [97,106] serves as a proof of concept of how designed collaborative play activities may offer opportunities to acquire practical skills and knowledge by working together and supporting each other. Through these experiences, individuals can build confidence in their abilities and support self-efficacy, essential for overcoming future challenges.

### Limitations

It should be noted that the sampling and conditions (eg, age range and size, cultural homogeneity) of this study are yet insufficient to generalize a broader claim. We also cannot conclude long-term effects of the game experience, as self-efficacy is a long-term construct that requires time and consistent influences. However, through the applied methodology and findings, we can already state how collaborative conditions within the gameplay support a relationship between players that might create opportunities for self-efficacy to be supported and applied in broader contexts.

### Implications

This study was developed within a DSR process [105], and one of the expected results is lessons for future design exercises that address similar concerns. As such, based on our findings, we propose a list of design principles for designing collaborative gameplay activities to support self-efficacy.

#### Adapting Dynamics of Collaboration

To develop a collaborative design, it was necessary to investigate collaborative processes, particularly the 3-level dynamics of collaboration [71]: co-construction, co-operation, and co-ordination. Understanding the characteristics of each level and the notion of transitions between levels led to the need to develop mechanisms that support them. In our game, this was achieved by having the shared player panel and associated actions, which create opportunities for player mutual support and prevention of breakdowns of the collaborative activity.

#### Creating a Common Goal

For effective collaboration, it is essential to share a common goal that fosters social interaction, communication, and collaboration among group members [45,53], integrating individuals’ prior knowledge and skills developed toward that joint objective. In our game, the colony’s survival as a collaborative goal results in the development of collective comprehension and knowledge based on each other’s ideas and thoughts (through vicarious experiences and verbal persuasion [12,13]), ultimately leading to problem-solving success. Furthermore, the pursuit of this common goal creates interdependence among group members, as the objective of the activity cannot be achieved alone [45,53]. Thus, all members must contribute collaboratively, recognizing that their own success depends on their peers.

#### Ways of Forming a Community

We suggest a game goal focused on a meerkat colony theme, in which players must collaborate to achieve it (co-construction level [71] - increase of the colony), establishing social norms, assuming diverse roles and actions in the game context (co-operation level [71]), cooperating to define them in the player panel, and distributing their coordinated execution among them (co-ordination level [71]). Such a collaboration context fosters a sense of community between the players, which is further supported by the following shared game elements and mechanics (inspired by the shared elements of [45,53]):

- Shared player panel: enables players to define actions they jointly decide to execute on the main board. This process was “necessarily” collaborative, as players had to discuss which actions (tasks) they wished to define (co-operation [71]), which in turn stimulated the construction of actions together for later execution (co-ordination [71]). This shared element also encouraged players to discuss strategies, offer assistance to one another, and engage in collaborative problem-solving. As players interact to define creature actions, they create an environment where verbal persuasion and vicarious experiences [12,13] play crucial roles. Through dialogue and observation of their peers’ actions, players gain insights into effective strategies and approaches to solving challenges. This collaborative approach may improve the performance of individual actions but also supports abstract problem-solving and decision-making skills related to CT activities [82-84]. By working together, players can leverage their collective knowledge and skills to overcome obstacles and achieve their goals within the game.
- Conjoined activities: develop tasks that individuals carry out (co-ordination [71]) to achieve the overall objective of the collective activity: for a group to collaborate effectively, each member must play a meaningful role in solving the problem, making a significant contribution to the group’s work.
- Shared in-game elements: design game actions that involve sharing elements, such as sharing a bag to increase food storage capacity. This will enable the players to collectively propose and decide their actions regarding those elements.
- Shared dice moves: explore the option to share values rolled by the dice to determine movements and actions on the main board, where players may choose to spend all or share action points with each dice roll. This mechanic provided situations where they would give up their resources for the benefit of another player’s movements and, consequently, the colony’s survival.
- Community’s evolution and shared fate: assume a community-wide goal throughout gameplay (in this case, the increase of colony members and their evolution from meerkat babies to adults), creating a shared sense of victory, joint rewards, and losses, strengthening collective success and fate.

These collaborative design elements and gameplay mechanics interconnected player activities and encouraged them to think together, communicate effectively, and coordinate their efforts, and above all, support each other’s experiences, subsequently creating opportunities for self-efficacy development regarding the skills required for in-game success.

### Conclusions

We introduce an innovative methodological approach on how to code and analyze the gameplay sessions to study how the design elements influenced collaborative interactions supporting self-efficacy. By following a qualitative content analysis and coding observations of the participants’ behaviors, it differs from previous studies focused on measuring aggregate results in self-efficacy [99-103] through pre- and postexperience questionnaires. The main contribution of this study, although related to improving learning science processes, is primarily a game design contribution, synthesizing a set of design principles resulting from the applied DSR [105]. By introducing gameplay experiences grounded on these design principles, these principles may inform future serious game design to be explored at an early age by designing collaborations to support self-efficacy related to STEM and other areas. Framing STEM promotion as a sociotechnical wicked problem [98], an interdisciplinary, long-term systemic transformation is required that mobilizes knowledge from diverse fields and design instruments capable of inducing social change. Our aim is that these design insights may be adopted and extended by other researchers in game design research and other fields to learn how to explore collaborative gameplay in other technical skill development contexts (eg, engineering, health, and education), potentially shaping academic interests and career paths in future generations.

## Supplementary material

10.2196/94416 Multimedia Appendix 1 Process conducted for this study, representing one round of a DSR cycle, the identified constructs from the literature from which the axial coding categories were retrieved for the content analysis, ending with the resulting design insights.

10.2196/94416 Multimedia Appendix 2 Questions representing the questionnaire that we followed post gameplay for the semistructured individual interviews, focusing first on the more global gameplay experience and suitability, focusing then on underlying self-efficacy perceptions. The questions were structured to retrieve the perceived effects of the four dimensions of self-efficacy.

## Abbreviations

CT: Computational Thinking
DSR: Design Science Research
GDPR: General Data Protection Regulation
STEM: Science, Technology, Engineering, and Mathematics
