# Supplementary material for: Collaborative Gameplay for Children’s Self-Efficacy in Computational Thinking: Qualitative Cross-Sectional Design Science Research Case Study
Source: JMIR Serious Games. 2026 Sep 9;14:e94416. doi: 10.2196/94416 (PMC13557326; doi:10.2196/94416)
Supplement: Supplementary file 1 [file games-v14-e94416-s002.docx]

## Interview Questions

The following questions represent the questionnaire that we followed post-gameplay for the semi-structured individual interviews, focusing first on the more global gameplay experience and suitability, focusing then on underlying self-efficacy perceptions. The questions were structured to retrieve the perceived effects of the four dimensions of self-efficacy.

**Gameplay experience:**

- Tell us which part of this game was the most fun;
- Tell us which part of this game was the most boring/least interesting;
- Tell us about a moment when you felt that your friend helped you.

**Game suitability:**

- Did you enjoy the game?;
- Do you think this game is for you?;
- Do you think this game would suit all of your classmates? Would they all enjoy it?

**Self-efficacy perceptions:**

- Can you tell me if the game becomes easier to play over time?
- Can you tell me if watching the other player help you?;
- Can you tell me if the other player’s support help you play the game?;
- Can you tell me if you are feeling happy? Do you think that helped you play the game?
